# Evaluation design of a reactivation care program to prevent functional loss in hospitalised elderly: A cohort study including a randomised controlled trial

**DOI:** 10.1186/1471-2318-11-36

**Published:** 2011-08-03

**Authors:** Kirsten JE Asmus-Szepesi, Paul L de Vreede, Anna P Nieboer, Jeroen DH van Wijngaarden, Ton JEM Bakker, Ewout W Steyerberg, Johan P Mackenbach

**Affiliations:** 1 Erasmus University Medical Centre, Department of Public Health, Rotterdam, the Netherlands; 2 Institute of Health Policy and Management, Erasmus University Rotterdam, the Netherlands; 3 ARGOS zorggroep, Schiedam, the Netherlands

## Abstract

**Background:**

Elderly persons admitted to the hospital are at risk for hospital related functional loss. This evaluation aims to compare the effects of different levels of (integrated) health intervention care programs on preventing hospital related functional loss among elderly patients by comparing a new intervention program to two usual care programs.

**Methods/Design:**

This study will include an effect, process and cost evaluation using a mixed methods design of quantitative and qualitative methods. Three hospitals in the Netherlands with different levels of integrated geriatric health care will be evaluated using a quasi-experimental study design. Data collection on outcomes will take place through a prospective cohort study, which will incorporate a nested randomised controlled trial to evaluate the effects of a stay at the centre for prevention and reactivation for patients with complex problems. The study population will consist of elderly persons (65 years or older) at risk for functional loss who are admitted to one of the three hospitals. Data is prospectively collected at time of hospital admission (T0), three months (T1), and twelve months (T2) after hospital admission. Patient and informal caregiver outcomes (e.g. health related quality of life, activities of daily living, burden of care, (re-) admission in hospital or nursing homes, mortality) as well as process measures (e.g. the cooperation and collaboration of multidisciplinary teams, patient and informal caregiver satisfaction with care) will be measured. A qualitative analysis will determine the fidelity of intervention implementation as well as provide further context and explanation for quantitative outcomes. Finally, costs will be determined from a societal viewpoint to allow for cost effectiveness calculations.

**Discussion:**

It is anticipated that higher levels of integrated hospital health care for at risk elderly will result in prevention of loss of functioning and loss of quality of life after hospital discharge as well as in lower burden of care and higher quality of life for informal caregivers. Ultimately, the results of this study may contribute to the implementation of a national integrated health care program to prevent hospital related functional loss among elderly patients.

**Trial registration:**

The Netherlands National Trial Register: NTR2317

## Background

Hospital admission is considered a risk (especially for older patients), which increases with age [1]. Among 70-year olds who are admitted in the hospital, 35% show functional loss at time of discharge when compared to the period before hospital admission, and this percentage rises as high as 65% for persons aged 90 years or older [2]. Hospital related functional loss among elderly is often associated with the risk of developing complications due to an illness or its treatment [1]. Nevertheless, functional loss among elderly persons is only partly the result of the patient's diagnosed illness at admission and treatment thereof [2], implicating that a hospital stay by itself leads to functional loss as well. Functional loss may lead to renewed hospital admission, prolonged hospital stay, admission in a nursing home or even early death [3,4]. Furthermore, it will lead to greater dependence, resulting in a higher burden of care for informal caregivers [2,5-7], higher utilization of professional health care and thus higher health care costs [8]. It is therefore important to prevent or reduce functional loss among elderly at an early stage [9].

Risk factors for functional loss are highly prevalent among elderly patients at time of hospital admission [10] and can be categorized into several domains: 1) physical status (e.g. age, functioning prior to admission to hospital, diagnosis, co-morbidities, low Body Mass Index/malnutrition, tendency to fall); 2) mental status (e.g. cognitive problems, delirium, depression, anxiety); 3) socio-economic situation (e.g. financial environment) and social environment (e.g. living arrangements prior to admission) as well as aspects regarding care such as poly pharmacy [2,5,8,11-21]. Even though functional loss is a recurrent problem among hospitalised elderly patients, hospital care is usually primarily focused on treating the medically diagnosed illness, thereby often neglecting reactivation care that may prevent functional loss. A "paralleled focused" treatment on reactivation treatment next to treatment of medical diagnosis may preserve functioning of the elderly patient at risk, thereby possibly maintaining quality of life and independence in activities of daily living in the period after discharge from the hospital, which in turn may lead to a lower burden of care for the informal caregivers of these patients as well as lower health care costs at a societal level. This article describes the study design (e.g. methods, setting, population, strengths, and weaknesses) of an evaluation study of a hospital based reactivation care program that is developed to provide reactivation care parallel with regular medical care. This new intervention program will be further referred to in this article as "the program" and will be compared to two control hospitals offering different levels of usual care.

### Description of the hospital based reactivation care program

The program is developed to prevent and/or reduce hospital related functional loss among at risk elderly by offering an individualized treatment plan that is based on problem-solving principles. It includes interventions that are integrated, multidisciplinary and goal-oriented at physical, social, and psychological domains of functional loss and combines existing treatment methods and routes for reactivating at risk elderly persons into an individual care package.

The program consists of several important elements: First, it aims to identify elderly persons at risk for functional loss at an early stage after hospital admission (= triage within 48 hours of admission). This will make early implementation of interventions possible, which may prevent functional decline and promote a quick return to independent living as well as preserve quality of life [7,22]. Second, the program consists of a combination of integrated interventions offered by a specialized multidisciplinary reactivation team with geriatric expertise. Based on existing literature, this approach is expected to lead to reductions in fall incidence, improved functioning, reduced length of hospital stay, lower (re)admissions to hospital and nursing homes, improved mental well-being of informal caregivers and higher perceived health and life satisfaction among patients as well as better coordination of treatment and follow up between different health care providers and finally, lower mortality [23-34]. Third, the multidisciplinary team uses Goal Attainment Scaling (GAS) to develop and monitor a personalized treatment plan. The GAS method has been successful in maintaining/improving functioning of elderly patients with complex health issues [35,36] and has been standardized for this population [37]. The GAS method consists of several phases: The multidisciplinary team identifies the baseline status of the patient and determines a goal. Then the team will monitor the development per patient and their informal care system by measuring progress regularly, making it possible to adjust interventions and/or goals when necessary. A final GAS measurement will take place to set up a follow up treatment plan before the patient is discharged to the home environment. The fourth element of the program is the possibility for patients with complex problems to be referred by the multidisciplinary team to a stay at a specialized centre for prevention and reactivation (further referred to in this article as "the reactivation centre" or "the centre"). The centre offers a combination of interventions aimed at improving a patient's ability to live as independently as possible in the home environment by providing extra-intensive thematic reactivation treatment alongside regular provisions. It includes specialized nursing home care, paramedical care, specialized mental health care, and treatment and consultations for primary informal caregivers if needed. Patients stay at the reactivation centre for a maximum of three months, after which the multidisciplinary team will develop an individual care-plan for follow up after discharge. Finally, the program will provide support to patients and their informal caregivers by means of a case manager with geriatric expertise who is involved in all aspects of care throughout the period of hospital stay as well as during the follow up period after hospital discharge (irrespective of a patient's destination after discharge whether this is their independent home, the centre for prevention and recovery, a nursing home or any other setting). The case manager is involved in identifying at risk patients in the hospital, coordinating the individual's care plan, coordinating follow up health care for a patient after discharge (e.g. in cooperation with general practitioner, home care or other first line care providers) and aims to support and motivate the patient in treatment adherence. In addition the case manager monitors a patient's risk factors for functional loss throughout all phases of care and may plan extra treatment if necessary as well as improve the care process where possible. Previous programs focused on case management or follow up care have lead to reductions in hospital admissions, nursing home admissions as well as a reduction in length of hospital stay [38,39]. Furthermore, case management may lead to improved access to health care, increased psychosocial support and improved communication with health professionals as valued by patients and their informal caregivers [40].

Even though earlier studies have shown the benefits of specialized multidisciplinary geriatric inpatient reactivation interventions (as well as similar programs for elderly persons living at home), insufficient data is available on programs offering a combination of abovementioned successful elements of care, their cost-effectiveness, and on how to define the patient group that benefits the most from these programs [39,41-45]. To our knowledge, the current evaluation of the new intervention program is the first to offer results on the effects of a combination of several successful elements of care as well as offer clear patient eligibility criteria for such an integrated program.

### Objectives of the evaluation

This evaluation study entails an effect, process and cost evaluation of offered geriatric health care and has four main objectives. First of all, the study aims to determine to what extent the program, in comparison with other usual forms of geriatric care, leads to a retention in functioning and quality of life of at risk elderly, a reduction in the burden of care for the patient's primary informal caregiver, shorter lengths of hospital stay, and a reduction of 'wrong bed' problems as well as (re-) admission to hospitals, nursing homes and mortality. In addition, it will show the extent to which program care including a stay at a reactivation centre, leads to better functioning and quality of life of elderly patients in need of complex care. Secondly, the study aims to determine to what extent the triage instruments used in the program detect increased risk for functional loss and to determine how criteria for triage should be adjusted to optimally link the offered interventions to the needs of the individual elderly at risk. Thirdly, the evaluation will determine to what extent the program, in comparison with other, usual forms of geriatric care in the Netherlands, leads to a better structure and process of care. Finally, the cost-effectiveness of the program (both including and excluding a stay at a reactivation centre) will be determined in comparison with other usual forms of geriatric hospital care in the Netherlands.

## Methods/Design

### Evaluation design

This evaluation study uses a concurrent mixed methods design (a combination of qualitative and quantitative research methods) to evaluate triage criteria, effects, processes and costs of the care provided in the three hospitals. It consists of a quasi-experimental study as well as a nested randomised controlled trial. Within the quasi-experimental study, the data collection of health care costs and outcomes on functional status and quality of life for patient and caregiver as well as other outcome measures such as cognitive functioning, duration of hospital stay and mortality of patients will be measured using a prospective cohort design. The "reactivation centre" component will be evaluated using a randomised controlled trial. Patients eligible for a stay at the reactivation centre will be randomised to program treatment with a stay at the centre or program treatment without a stay at the centre. The effects of a stay in the reactivation centre will be measured at three months after hospital admission and the effects of a centre stay in combination with program aftercare at home will be evaluated at twelve months after hospital admission. In addition, a set of quantitative process indicators will be collected both for program treatment with and without a stay at the centre (e.g. which disciplines were involved in treatment, how soon after admission the treatment started). For an in-depth evaluation of the effects of the program, data is collected on the differences in the healthcare processes between the three hospitals as well as differences in offered follow up care between the three hospitals. Qualitative data will be collected through interviews, observations and document analysis at similar times as the effect evaluation. These qualitative measures will support the comparison of the quality of care processes between the three hospitals.

The study protocol was approved by the medical ethics committee of the Erasmus Medical Centre, Rotterdam, the Netherlands, under protocol number MEC2011-041.

### Setting

Three hospitals with different levels of geriatric care will be compared in this evaluation. The first hospital (Ruwaard van Putten, Spijkenisse) offers care without clinical geriatrics, with hospital replacement care through a care hotel and no follow up in primary care. The second hospital (St. Franciscus Gasthuis, Rotterdam) offers care with coordinated discharge and hospital replacement care (through care hotel "Aafje") and without follow up in primary care. The third hospital (Vlietland + Argos Zorggroep, Nieuwe Waterweg, Noord) is the intervention hospital and offers the new hospital based reactivation care program which includes clinical geriatrics, intensive reactivation care after hospital stay (through a stay at the centre for prevention and reactivation) and with follow up in primary care (through case management). The three hospitals have been chosen as they are similar in patient case mix as well as offer geriatric care in different dosages and with different elements of care.

### Pilot study

**Figure 1:**
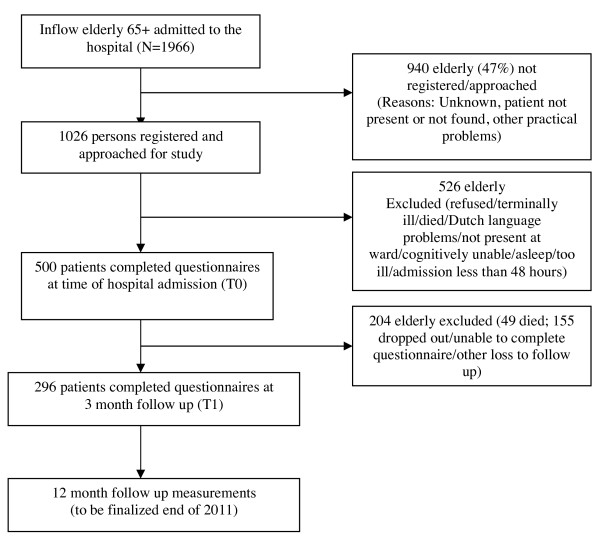
**Flow chart pilot study**.

A pilot study was conducted in the intervention hospital (Vlietland hospital) to choose the best triage instruments to identify elderly patients eligible for the reactivation program. Furthermore, the pilot results will be used to identify possible practical implementation problems in preparation for the main evaluation study and serve as base for power calculations for the main study. In the pilot study, all patients (65 years or older) who were admitted to the Vlietland hospital between June 2010 and October 2010, were asked to participate. Around 500 patients and 200 informal caregivers were included at baseline (within 48 hours after hospital admission) and of this group around 300 patients and 160 informal caregivers completed questionnaires at the 3-month follow up (see figure 1: Flow chart pilot study). Follow up measurements at twelve months after hospital admission will be finalized in November/December 2011.

### Participants

#### Population

**Figure 2:**
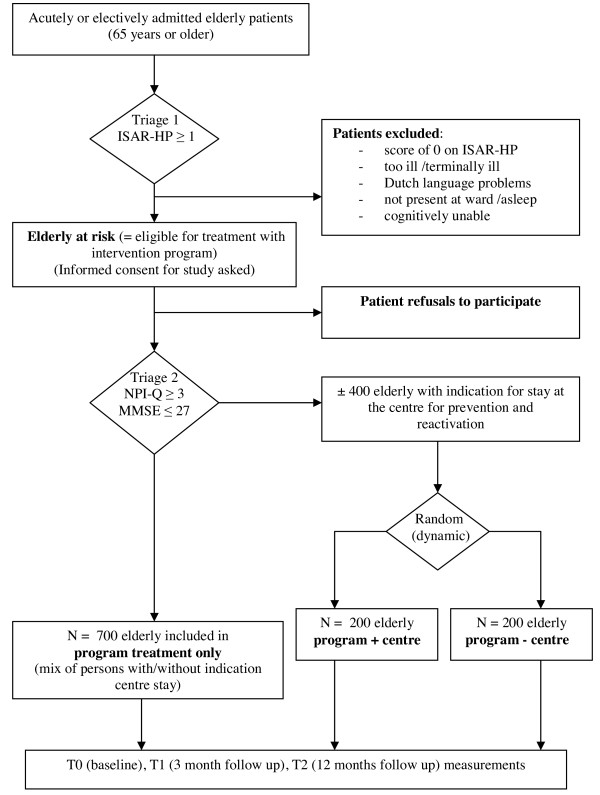
**Flow chart intervention hospital**.

**Figure 3:**
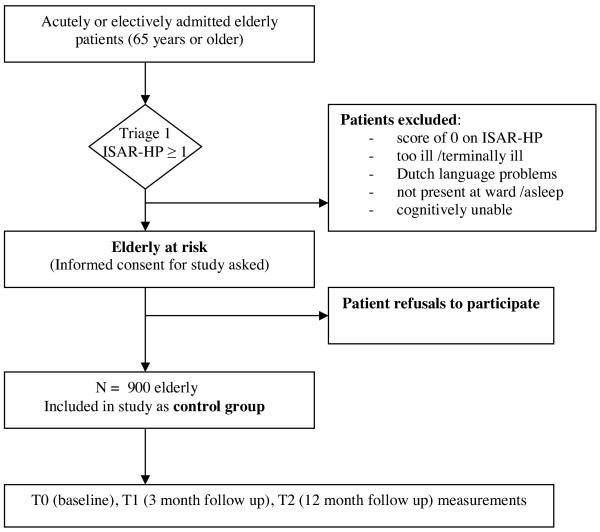
**Flow chart control hospitals**.

The target population of the study consists of elderly persons (65 years and older) who are at risk for functional loss and are admitted to one of the three hospitals for at least two days. All patients will receive the usual care offered by each of the hospitals. All relevant hospital departments will be included in the study and admission may be elective or acute. Figures 2 and 3 (flowcharts) provide an overview of the flow of patients in the study. Through a first triage step, all patients at risk for functional loss will be identified with the ISAR-HP [46,47]. These at risk patients will be eligible to receive the hospital based reactivation care program and therefore will be asked to participate in the study. In the Vlietland hospital, an additional triage will take place using the short Neuropsychiatric Inventory [48,49] and the Mini Mental State Examination [50] to identify elderly patients eligible for a stay at the reactivation centre. This group will then be randomised to program care including a stay at the centre (n = 200) or program care excluding a stay at the centre (n = 200). The primary informal caregivers of the participating patients will be asked to answer several questions about the patient in a telephone interview as well as fill out mailed paper questionnaires on quality of life, burden of care and other outcomes at time of hospital admission of the patient, three months after hospital admission and twelve months after hospital admission. Furthermore, health care professionals from each participating hospital will be asked to complete a survey on processes of health care.

#### Inclusion criteria

• patients aged 65 years or older

• admitted in one of the participating hospitals and staying >48 hours

• at risk for functional loss (ISAR HP ≥ 1)

Additional criteria for a stay at the centre for prevention and recovery

• ISAR HP ≥ 2 and/or MMSE ≤ 27 and/or NPI ≥ 3

#### Exclusion criteria

• Unable to answer questions or follow instructions (e.g. due to severe cognitive problems (MMSE score <12/delirium/coma) within 48 hours of admission in the hospital

• Not able to understand the Dutch language

• Life expectancy <3 months.

#### Power calculation and effect size

For the prospective cohort we expect to be able to collect a sample size of around 1100 patients in the intervention hospital (900 patients treated with the new intervention program and 200 patients treated with the new intervention program including a stay at the reactivation centre). Samples of minimal 500 to 600 patients will be collected in each of the two control hospitals. These estimations are based on the average number of elderly patients who are admitted to the different hospitals during our inclusion period of one year. According to preliminary pilot results on activities of daily living (Katz-15 ADL score), a population of n = 500 in the control hospitals will lead to around n = 300 persons analyzable at three months, whereas a baseline population of n = 1100 in the intervention hospital will lead to around 733 persons analyzable at three months. Using an effect size of 0.25 this will lead to a power of 95% [46]. Furthermore, to detect a smaller effect size (Cohen's D of 0.2), n = 1100 in the intervention hospital and n = 500 for the control hospitals will lead to a power of 83%. If possible, we will aim for a larger sample size in the control hospitals than the expected n = 500, (preferably 900), which will lead to N = 733 analyzable in the intervention hospital versus n = 600 analyzable in the control hospitals, with an effect size of 0.2 leading to a power of 95%. Abovementioned sample sizes are large enough to allow for reliable analysis per subgroup (e.g. subgroup of specific diagnoses) and sets of risk factors.

#### Randomisation

Dynamic randomisation will be used to select patients who receive the new program treatment. It is estimated that the population of patients eligible for the program will be higher than the actual amount of patients that can be treated with the program due to restrictions on available personnel, materials and budget. Therefore, randomisation criteria will change dependent on what is logistically possible (= dynamic). Since the reactivation centre has a maximum capacity of 200 patients per year at this time, dynamic randomisation will be carried out by computer where the chance of referral to the program and a stay at the centre will be reduced accordingly as fewer resources are available to provide care and fewer places in the centre are available.

#### Blinding

Treatment allocation is by definition un-blinded, but since the hospital based reactivation care program is in fact the usual care provided in the intervention hospital it is possible to maximize blinding of data collectors by describing the three offered health care programs as usual care in all communications, thereby concealing treatment allocation. Furthermore, blinded analyses of data will take place when possible.

### Data collection

There is no clear consensus on the time period during which effects of interventions on physical functioning and quality of life will be maintained. In some studies effects were present at six to twelve months after the start of the intervention, with the largest effect present around three months [24,26]. Therefore, main data collection of patient and caregiver outcomes takes place at time of hospital admission (T0), three months after hospital admission (T1) and twelve months after hospital admission (T2). Trained research nurses and students will administer questionnaires to patients by means of interviews at T0, T1 and T2. Furthermore, informal caregivers will receive paper questionnaires sent by mail. Patient and caregiver outcome measures will then be compared between the three hospitals. In addition a survey is administered among personnel of the three hospitals at one time during the second half of the inclusion period. Additional triage, outcomes, process, and cost information is collected from patient files and hospital ICT systems at time of discharge, during the intervention and in retrospect. Based on the pilot results we expect around 60% of patients analysable at 3 month follow up. Loss to follow up is minimized by making house calls and interviewing the patients in person [51]. Tables 1, 2, 3, 4 and 5 show a complete overview of outcome variables and data collection instruments for demographics and triage (table 1), effect evaluation (tables 2 and 3), process evaluation (table 4) and cost evaluation (table 5).

**Table 1:** Data collection of patient and informal caregiver: demographics and triage

Evaluation	Variables and+ instruments	Data collection methods	Data collection times			**Notes**:
			**T0**	**T1**	**T2**	

**General**						
Patient	**Demographics**					
	MDS (age, ethnicity, SES, education)	Interview patient	**X**	**X**	**X**	Partly at T1 and T2
		Patient hospital files	**X**			incl info hospital ICT
Informal caregiver	**Demographics**					
	MDS (relation to patient, SES etc)	Mailed paper questionnaire	**X**	**X**	**X**	Partly at T1 and T2

**Triage**						
Patient	**Risk for functional loss**					
	ISAR-HP	Interview patient	**X**			
	MMSE (cognitive functioning)	Interview patient	**X**			
	NPI-Q (neuro-psychiatric functioning)	Phone interview informal caregiver	**X**			

* T0 = within 48 hours of hospital admission; T1 = 3 months after hospital admission; T2 = 12 months after hospital admission; MDS = Minimal Data Set; ISAR-HP = Identification Seniors At Risk (Hospitalized Patients); MMSE = Mini Mental State Examination; NPI-Q = Neuro-psychiatric Index;

**Table 2:** Effect evaluation: Data collection of patient outcome variables

Patient outcome variables + instruments	Data collection methods	Data collection times			**Notes**:
		**T0**	**T1**	**T2**	

**Quality of life**					
SF-20	Interview patient	**X**	**X**	**X**	5 items part of MDS
EQ-5D (6D)	Interview patient	**X**	**X**	**X**	Part of MDS
SPF_IL	Interview patient		**X**	**X**	
**Physical performance**					
Katz -15	Interview patient	**X**	**X**	**X**	Part of MDS
Short Physical Performance Battery	"Do" test patient		**X**	**X**	
LAPAQ (physical activity)	Interview patient		**X**	**X**	
**Cognitive/psycho/social performance**					
NPI (neuro-psychiatric functioning)	Interview caregiver	**X**	**X**	**X**	
MMSE (cognitive functioning)	Interview patient	**X**	**X**	**X**	Short MMSE (T1 + T2)
Geriatric Depression Scale (depression)	Interview patient	**X**	**X**	**X**	
Global Deterioration Scale (dementia)	Phone interview inf. caregiver	**X**	**X**	**X**	
Loneliness scale Gierveld (social)	Interview patient	**X**			
**Intramural Residence**	Medical registries		**X**		
**(Re-)admission hospital/nursing home**	Interview patient/caregiver		**X**	**X**	
**Mortality**	Medical registries		**X**	**X**	
**Patient self management**					
SMA-S	Interview patient		**X**	**X**	

*T0 = within 48 hours of hospital admission; T1 = 3 months after hospital admission; T2 = 12 months after hospital admission; * MDS = Minimal Data Set; SF-20 = Short Form 20; EQ6D = EuroQol; SPF-IL = Social Production Function questionnaire; SPPB = Short Physical Performance Battery; LAPAQ = LASA physical activity questionnaire; MMSE = Mini Mental State Examination; NPI = neuro-psychiatric index;; GeDS = Geriatric Depression Scale; GloDS = Global Deterioration Scale;; SMA-S = Self Management Ability Scale.

**Table 3:** Effect evaluation: Data collection of informal caregiver outcome variables

Informal caregiver outcome variables + instruments	Data collection methods	Data collection times			Notes
		**T0**	**T1**	**T2**	

**Quality of life**					
EQ-6D	Mailed paper questionnaire	**X**	**X**	**X**	Part of MDS
SF20	Mailed paper questionnaire	**X**	**X**	**X**	5 items part of MDS
Carer QoL	Mailed paper questionnaire	**X**	**X**	**X**	Part of MDS
**Burden of care**					
ARS (objective)	Mailed paper questionnaire	**X**	**X**	**X**	Part of MDS
Questions on time spent on care tasks	Mailed paper questionnaire	**X**	**X**	**X**	
CSI (subjective)	Mailed paper questionnaire	**X**	**X**	**X**	
SRBS (subjective)	Mailed paper questionnaire	**X**	**X**	**X**	Part of MDS

*T0 = within 48 hours of hospital admission; T1 = 3 months after hospital admission; T2 = 12 months after hospital admission; * MDS = Minimal Data Set; SF-20 = Short Form 20; EQ6D = EuroQol; GeDS = Geriatric Depression Scale;; ARS = Activity restriction scale; CSI = Caregiver strain index; SRBS = Self-rated burden scale.

**Table 4:** Process evaluation: Data collection of process outcome variables

Outcome variables and instruments	Data collection methods	Data collection times			Notes
		**T0**	**T1**	**T2**	

**Patient experiences with quality of care**					
PACIC	Interview patient		**X**	**X**	
(H)CAPHS	Interview patient		**X**		
**Caregiver experiences with quality of care**					
SASC - adapted for elderly care (+ own formulation additional questions)	Mailed paper questionnaire			**X**	
**Process of care delivery hospital**					
Process indicators (own formulation)	Medical registers	**X**			+ time after hospital discharge
**Process of care delivery professional view**					
TCI (+ own formulation additional questions)	Mailed paper questionnaire				Last months inclusion period
ACIC (only partly)	Mailed paper questionnaire				Last months inclusion period
RCSP	Mailed paper questionnaire				Last months inclusion period

* T0 = within 48 hours of hospital admission; T1 = 3 months after hospital admission; T2 = 12 months after hospital admission * PACIC = Patient Assessment of Chronic Illness Care; (H)CAPHS = (Hospital) Consumer Assessment of Healthcare Providers and Systems; SASC = Satisfaction with Stroke Care Questionnaire; TCI = Team Climate Inventory; ACIC = Assessment of Chronic Illness Care; RCSP = Relational Coordination Survey for Professionals.

**Table 5:** Cost effectiveness evaluation: Data collection health care volumes and prices

Outcome variables	Data collection methods	Data collection times			Notes
		**T0**	**T1**	**T2**	

**Health care volumes**					
Period hospital admission:	Hospital registries	Time of hospital discharge			+ during inclusion
*- amount of hospital days (days admitted)*	Hospital registries	Time of hospital discharge			
*- total days/time admitted at ICU*	Hospital registries	Time of hospital discharge			
*- amount of consults medical/paramedical*	Hospital registries	Time of hospital discharge			
*- amount of large scans and diagnostics*	Hospital registries	Time of hospital discharge			
*- amount of large treatments/operations*	Hospital registries	Time of hospital discharge			
*- wrong bed days*	Hospital registries	Time of hospital discharge			
*- amount of days admitted*	Hospital registries	Time of hospital discharge			
*- amount of consults medical/paramedical*	Hospital registries, interviews personnel	Time of hospital discharge			+ during inclusion
*- time/amount of multidisciplinary meetings*	Hospital registries, interviews personnel	Time of hospital discharge			+ during inclusion
Health care utilization patient in periods before and after hospital stay	Interview patient and mailed paper questionnaire primary caregiver	**X**	**X**	**X**	
*- homecare/nursing home/etc*					
Average time per consult	Interviews professionals (sample)	During inclusion period			
Amount informal care	MDS + questionnaire informal caregiver	**X**	**X**	**X**	Amount in hours
Amount (multidisciplinary) coordination	Interviews professionals				Amount in hours
**Health care prices**					
price per hospital day	DBC information + manual cost research	Retrospective			Similar calculations for prices hospital, CPH, care hotels etc.
price per day/hour admitted at ICU	DBC information + manual cost research				
price of consults medical/paramedical	DBC information + manual cost research				
price of large scans and diagnostics	DBC information + manual cost research				
price of large treatments/operations	DBC information + manual cost research				
costs of home care/other care	Integral costs per product cluster	Retrospective			
costs informal care by primary caregiver	Market price/missed wages & time	Retrospective			
travel costs (average)	Literature	Retrospective			

*T0 = within 48 hours of hospital admission; T1 = 3 months after hospital admission; T2 = 12 months after hospital admission.

#### Demographics and triage (table 1)

##### Triage: identification of elderly at risk for functional loss

Triage data is gathered within 48 hours of hospital admission in order to identify elderly at risk for functional loss at an early stage. Based on the pilot results the Identification of Seniors At Risk-hospitalized patients (ISAR-HP) is chosen as the main triage instrument in order to achieve information on a combination of factors that have shown to be important in predicting functional loss. The ISAR HP questionnaire is administered to the patient and consists of four questions on education and need of help with travelling, walking or help with housekeeping in the period before admission to the hospital [46]. A person is considered at risk for functional loss and is therefore eligible for treatment with the program if he/she scores one or higher on the ISAR HP. This is a different score from the originally set cut off score of 2 or higher as is stated in the paper written by Buurman et al (2010), and can be explained by the differences in characteristics and diagnoses of the studied population. In addition, the NPI-Q and MMSE are administered in order to identify elderly who are eligible for an additional stay at the centre for prevention and reactivation as part of the program. Patients will be considered eligible for a stay at the reactivation centre when they score 2 or higher on the ISAR HP and/or 3 or higher on the NPI-Q and/or 27 or lower on the MMSE. The Neuropsychiatric index (NPI-Q) is the validated short version of the NPI [49]. It aims to identify neuropsychiatric symptoms present in the patient in the last month by means of twelve symptoms (e.g. delusions, aggression, hallucinations) and also measures the emotional burden for the caregiver. The NPI is administered to the primary informal caregiver of the patient by means of a telephone interview at time of hospital admission. The Mini Mental State Examination (MMSE) measures cognitive functioning by means of interviewing the patient using questions on orientation in time and space, short-term and middle-term memory, comprehension and other cognitive dimensions [50,52].

##### Demographics

Data on demographics (e.g. age, socioeconomic status, marital status, and gender) is gathered at T0, T1 and T2 by means of the Minimal Data Set (MDS) and hospital registries. The MDS is developed in light of the national program elderly care in the Netherlands and aims to compare elderly persons as well as their caregivers participating in different projects in the Netherlands by measuring demographics, quality of life, ADL functioning, experienced health and health care utilization (patient-level) as well as demographics, experienced health and burden of care, quality of life, and objective burden of care (informal caregiver level). The MDS is a combination of (parts of) validated questionnaires and is administered by trained research nurses and trained students (e.g. medical students or other students who have experience with research and/or elderly care), who interview patients at T0, T1 and T2. The data is collected from informal caregivers by means of mailed paper questionnaires, which are self-administered by informal caregivers and then sent back to researchers. A reminder including an extra copy of the questionnaire is sent to informal caregivers if they did not send back the first questionnaire. Additional data on demographics as well as data for other elements of the evaluation (e.g. medication, diagnosis, specialist consults) is collected from medical registries after hospital discharge.

#### Effect evaluation (tables 2 and 3)

All outcome data of the patient are collected by the same means as the MDS described above.

##### Quality of life (patient)

The EuroQol (EQ6D) is administered to measure quality of life among patients and their caregivers. It is part of the MDS and will be used to calculate cost-utilities of health care [53].

The Dutch version of the SF-20 is administered and aims to score 6 sub-dimensions such as physical functioning, social functioning and experienced health [54]. The SF-20 is chosen since it is quick to administer and many of its questions are already part of the MDS. The SF-20 has shown good test-retest reliability and acceptable convergent and discriminative validity for a group of elderly persons, even though some precaution is advised in using the questionnaire with elderly people living at home [55]. The short version of the Social Production Function Scale SPF-IL scale measures social well-being by means of the dimensions "affection", "behaviour confirmation" and "status" as well as physical well-being by means of the dimensions "comfort" and "stimulation" [56].

##### Physical functioning (patient)

The Katz-15 index of activities of daily living measures function over time by means of questions on several domains such as bathing, dressing, toileting, transferring, continence and feeding [57,58]. The LAPAQ (LASA Physical Activity Questionnaire) is an interview administered questionnaire measuring frequency and duration of activities such as household activities, walking, gardening and sports [59]. The SSPB (short physical performance battery) is an objective physical performance test consisting of repeated chair stands (number of stands and amount of time standing), balance testing (three different stands), and walking (2.44 meters). This test is necessary in order to see if the results from the subjective physical performance tests are in agreement with measured objective physical capabilities [60,61].

##### Cognitive and neuropsychiatric functioning (patient)

The NPI and MMSE (see triage for explanation) are administered to measure cognitive and neuro-psychiatric functioning over time, with the short version of the MMSE being administered at follow up instead of the longer version that was administered at time of hospital admission. Nevertheless, the results are still comparable using existing and tested transformation scores. The Global Deterioration Scale (GDS) measures a patient's stage of dementia by means of 7 levels, from 1 (normal functioning) to 7 (very serious dementia) and is administered to the informal caregiver of the patient [62].

##### Social and psychological functioning (patient)

The Geriatric Depression Scale (GDS-15) identifies and measures functional as well as mood symptoms of depression [63]. The GDS-15 has been validated in geriatric inpatients as well as in primary care and community-living elderly [64,65]. The Loneliness scale consists of 11 questions and measures social functioning of the patient [66].

##### Self-management (patient)

The SMA-S (Self Management Ability Scale) measures the ability of a person to manage his/her own general daily life activities in the past months. It contains items on several subjects such as activities the patient initiates; activities the patient starts now but expects to benefit from later; general activities; combining activities; the success or failure of activities; and dealing with adverse experiences [67].

##### Quality of life (caregiver)

The carer quality of life questionnaire (Carer QoL) measures quality of life of caregivers and is part of the MDS [68]. The EuroQol is also administered to the caregiver as part of the MDS (see Quality of Life patients).

##### Burden of care (caregiver)

Objective burden of care for the caregiver is measured using the Activity Restriction Scale [69] and additional questions on objective burden of care [70,71]. Subjective burden of care is measured with the Self Rated Burden Scale and Caregiver Strain Index or CSI [72,73].

#### Process evaluation (table 4)

The process evaluation will look at process indicators thereby showing the extent to which the new program leads to better structure and process of care in comparison with other usual forms of geriatric care in two other hospitals in the same region (e.g. improvements in coordination between care-providers, patient logistics, information logistics and support). In addition, the process evaluation will focus on how and to what extent the program is actually implemented according to plan. This requires measuring instruments that are sensitive for specific interventions and which are connected with the expected alternations in the outcomes of care for the elderly and caregiver. In order to do this, sub-domains of the care process from a patient, caregiver and professional point of view will be measured. In addition, qualitative data is gathered to explain quantitative outcomes. Described processes and provided interventions will be linked to outcomes in order to provide a complete description of the evaluation of this transition project.

##### Process of care (patients)

Patient experiences with delivered care are measured at T1 by means of the Patient Assessment of Chronic Illness Care (PACIC) questionnaire, and consists of questions on care received in the last 3 months [74]. In addition, specific experiences with hospital care delivered during total hospital stay around T0 are measured with the (Hospital) Consumer Assessment of Healthcare Providers and Systems ((H)CAHPS), which consists of questions on treatment by nurses and doctors, hospital environment, experiences with hospital stay as well as discharge from the hospital, and general appreciation for the hospital [75]. Registering process indicators involves a continuous measurement of the care provided to the elderly and their caregiver. At this time it does not appear possible to make use of an Electronic Patient Dossier (EPD) in all three hospitals. Therefore, process indicators will be collected partly from existing registrations. The remaining indicators are collected by research nurses and students. Insight into the care process is provided by covering the topics of 'determining vulnerability'; 'provided medical care (diagnostics and treatments)' and 'the extent of multidisciplinary meetings'.

##### Experiences quality of care (caregiver)

The SASC (Satisfaction with Stroke Care Questionnaire) originally measures patient satisfaction with stroke care. For current research, it has been adapted to caregiver satisfaction with the care for vulnerable elderly after discharge. It covers subjects on both the acute and chronic phase of care: experienced caregiver respect and information provision during elderly hospital stay; the amount of caregiver support and information provision after elderly discharge [76,77]..

##### Process of care (professional)

The TCI (Team Climate Inventory) has been used as an improvement tool for assessing team function to identify areas that could be improved. It contains 14 items on several team dimensions such as: task orientation and support for innovation [78]. The ACIC (Assessment of Chronic Illness Care) is a practical quality-improvement tool to evaluate the delivery of care for chronic illness in six areas: community linkages; self-management support; decision support; delivery system design; information systems and organisation of care [79]. The RCSP (Relational Coordination Survey for Professionals) measures the relational dynamics of coordinating work. The self-administered questionnaire contains 8 items. Different professionals are asked how frequently, timely and accurately they solve problems and share goals with other professionals while treating vulnerable elderly [80].

##### Qualitative process measures

Qualitative measures will complement the quantitative data collection, thereby strengthening the study design by providing the mixed method component mentioned earlier. Qualitative measures will provide additional in-depth information on the context in which the implementation of care and interventions takes place (structure). An audit study based on expert opinion and literature will provide information on general quality of care (e.g. by means of showing/evaluating differences and similarities in the care and interventions that are provided in the three hospitals). Furthermore, a fidelity study will collect information on the differences between planned and actual implementation of care and interventions in the hospitals (is care implemented as it should be by the professionals?). Finally, case studies by means of qualitative interviews with professionals as well as observations within the hospitals will provide further information and insight as they provide a context in which quantitative outcomes can be placed.

#### Cost-effectiveness evaluation (table 5)

A cost-utility analysis will compare cost differences (incremental costs) of provided health care in the three hospitals with the difference in health effects measured in quality adjusted life years (QALYs). QALYs combine alterations in quantity and quality of life (mortality and morbidity) into a combined generic instrument. Data on quality of life will be measured by means of the EQ6D and SF20 for patients and by means of the EQ6D, CarerQoL and SF-20 for the primary informal caregiver (see effect evaluation). Cost information is gathered by means of hospital information systems, patient files and questionnaires for both patient and primary caregiver at baseline (T0), after three months (T1) and after 12 months (T2). A societal perspective is used; taking into account both direct and indirect costs within as well as outside health care. Data collection on utilization of care in hospital (nursing days, diagnostic and therapeutic activities and out-patient visits), nursing home (days), rehabilitation (admissions/outpatient), and home care (care hours according to product clusters) will take place centrally, according to a standard method, by means of standardized files and standard cost diaries as well as through patient and caregiver questionnaires

### Data analysis

#### Triage

Data will be gathered on risk factors for functional decline and predictive models for functional decline at 3 and 12 months can be developed using multivariate regression techniques and data from control settings. The quality of the predictive models will be assessed by explained variance for continuous outcome measures and by ROC-curves for dichotomous outcome measures. The quality of the model that fits the program eligibility criteria will be compared to models with other risk factors involved and/or other cut-off points on the triage instruments. This way the added value of another triage method can be objectively assessed. These analyses were already partly done using preliminary pilot study results but will be repeated using final results of the main evaluation study. Furthermore, main study results will be used to evaluate the validity of the model(s) with internal validation techniques (bootstrapping) and cross validation between the three settings whereby each setting acts as a test-population for model(s) developed in the other settings [81].

#### Effect evaluation and process evaluation

##### Effects and process evaluation of the hospital based reactivation care program

Corrections will take place by means of 'analysis of covariance' for baseline differences in determinants between the locations that could explain differences in functioning or quality of life. Multiple regression analysis will be administered for various outcome variables such as linear regression for continuous outcomes; logistic regression for dichotomous outcomes; proportional odds regression for orderly outcomes and Cox proportional hazards regression for events that occur over time (such as death). Analysis of outcomes at three and twelve months will take into account dependency of these outcomes within persons. The degree of exposure to integrated, multidisciplinary care within the intervention location (process evaluation) will also be correlated with effect measurements in order to see whether greater exposure leads to greater effects. Regression analysis will trace sub-group effects and an interaction term will be included in the model, between type of hospital and sub group (e.g. elderly at high risk for functional loss in comparison to elderly at relatively lower risk for functional loss).

##### Effects and process evaluation of the centre for prevention and recovery

A randomised controlled trial will be analyzed according to the "intention to treat" principle. The process evaluation will study which treatments are carried out in exactly which way. Since differences in case mix and treatment regiments that differ from the program can confound the relation between the program and its expected effects, analysis will be corrected for these possible confounders. For example, data regarding diagnoses of elderly at admission and discharge will be collected and quantitative, clinical treatment data is collected during intake which is focused on medical diagnosis in order to correct for differences in treatment regiments. The randomisation leads to balance between arms of the randomised controlled trial in observed and unobserved predictors of functional decline. Important baseline characteristics are taken into account in analyses to correct for imbalance that might occur by coincidence, thereby increasing power.

#### Cost-effectiveness evaluation

Primary outcome measure is costs per QALY. A cost-utility analysis will compare cost differences (incremental costs) with the difference in health effects measured in QALYs. In order to calculate costs, the volume of care will be linked to the actual, integrated cost prices per medical service [82]. Net costs per nursing day will be calculated as well as the costs for diagnostics and therapy with help from the manual of cost research in economic evaluations [83] and will be judged for usability according to recent DBC-information. Wrong bed days will be estimated according to the method of Van Straten [84]. The extra costs of a stay at the centre (e.g. training, availability specialist care doctors and nurses, mobilization, extra physiotherapy etc) in comparison with the usual geriatric care provided in the program will be measured, making a distinction between once-only costs and structural costs [85]. Integrated costs per day nursed at the centre will be calculated with aid of the activity base costing method [82]. Data on personnel, material costs, diet-related costs, accommodation and overheads will be accessed using centre registries and information systems, and extra information is collected through regular observation and self-registration of professional activities. Integrated costs per hour, per product cluster, will be used for home-care. For the remaining extramural care (general practitioner care, physiotherapy, social services etc.) costs will be assessed with information from cost manual and recent cost-price research. Costs are discounted at a constant discount rate of 4% per year. Future health effects are discounted at a constant discount ratio of 1.5% per year. Net savings could occur on balance during hospital care, whilst a stay at the reactivation centre as well as home care will lead to use of additional means. This valance of savings and extra costs cannot be indicated in advance. It is expected that continuity of care will possibly lead to considerable savings [86].

## Discussion

The aim of this evaluation study is to compare outcomes, processes and cost effectiveness of three different healthcare programs provided for "at risk" elderly whereby a new 'hospital based reactivation care program' in the intervention hospital will be compared to the usual care provided in two control hospitals.

### Strengths

First of all, this study uses a mixed methods design of quantitative and qualitative measures that provide information on three elements as stated by Donabedian [87]: structural issues (e.g. materials, personnel, organization and coordination of care), processes (e.g. activities of professional in diagnosing and treating the patient) and patient and caregiver outcomes such as physical functioning and quality of life. According to Donabedian, the combination of these elements allows for better interpretation of findings, as one method may strengthen interpretation in cases where another method cannot explain variances or outcomes. Qualitative data may generate further hypotheses that may be explained by quantitative process and outcome data. It can also provide a context within which outcomes can be explained more in-depth. Secondly, a pilot study was conducted at the intervention hospital before implementing the program, which optimised beforehand the triage for selecting patients to be included in the main study as well as the power calculations and the practical implementation of the main study. Several practical and implementation problems were encountered in the pilot study (e.g. logistics within hospital, personal communication with hospital personnel etc), making it possible to prevent similar problems and sources of bias when conducting the main study. Within the cohort study, dynamic randomisation will be implemented in order to prevent extra bias and missing data per questionnaire. Furthermore, loss to follow up will be minimized by conducting personal interviews through house calls at three months and 12 months after hospital admission. Finally, the evaluation will be conducting a cost-effectiveness study, which will improve/increase current knowledge on the feasibility of implementing transition programs such as the one evaluated here.

### Weaknesses

This study is mainly a cohort study and not a randomised controlled trial (except for the evaluation of a stay at the centre for prevention and reactivation). Nevertheless a cohort study seems our best option for several reasons: Firstly, during hospital treatment, contamination of a control group would be inevitable within one hospital since the same personnel will be treating patients from different groups at the same departments [88]. Secondly, the new program will be the standard care provided in the intervention hospital thereby making randomisation within the hospital not possible. Furthermore, randomisation of treatments between hospitals is not possible since each hospital already has its own standard provided care. Thirdly, during the follow up period after hospital discharge people already have their own regular general practitioner (GP) whose practice is usually close to the patient's home and who is familiar with the patients health and history, making it unrealistic to randomize patients among GP's [88] or home care organizations as well as other first line practitioners. Finally, by conducting a prospective cohort study we aim to investigate health care as provided in a real life situation, thereby improving the generalisability of the study.

Another possible weakness of this study might be the fact that transitions within the three hospitals unrelated to the study may influence outcomes (e.g. at the time of writing this protocol plans exist for starting up specialized clinical geriatric care in the St. Franciscus Gasthuis in light of implementation of national guidelines on elderly care). This may alter differences in levels of health care provided by the hospitals over time thereby influencing outcome and process results. Nevertheless, these changes reflect how health care transitions evolve in real life situations, making the outcomes very valuable nonetheless. They will therefore be monitored closely by means of our quantitative and qualitative process evaluation. To describe the program we will use a methodological approach that combines qualitative and quantitative (mixed) research methods, enabling a thorough and comprehensive evaluation of care for elderly at risk for hospital related functional loss. The introduction of complex, multi component interventions such as this program is sensitive to an array of influences such as details of implementation and context [89,90] and as such calls for embracing a wide range of scientific methodologies. Such a wide range of scientific methodologies helps to obtain information on both mechanisms and contexts, adds to the knowledge on the feasibility and costs of different forms of integrated health care, and highlights the factors that are likely to influence the success and failure of integrated health care for elderly at risk for hospital related functional loss.

### Clinical implications

The results of the study will help determine the most effective way of identifying and treating at risk elderly who are admitted in the hospital in order to prevent unnecessary hospital related functional loss among this group and keep them as independent as possible for as long as possible after they are discharged. In addition, the study may show effective ways to lower the burden of care for primary informal caregivers of elderly patients at risk for functional loss as well as improve their quality of life. Furthermore, the results will increase knowledge on practical issues of implementing a transition in health care and on ways to improve coordination between first and second line care.

### Research implications

By comparing costs, effects and processes of different levels of integrated health care programmes offered in three hospitals, this study will extend our knowledge on how to prevent hospital related functional loss among at-risk elderly patients in a more cost effective way. This in turn may lead to further research on creating and evaluating similar (improved) integrated health care programs thereby strengthening the health care offered to elderly patients at risk for hospital related functional loss at both a regional and national level.

## Competing interests

The authors declare that they have no competing interests.

## Authors' contributions

KA drafted the manuscript as well as the original METC protocol. PdV, project coordinator, edited and reviewed the manuscript and finalized the original METC protocol. JM, project leader, was responsible for the original final proposal and obtaining funding for this study. AN and ES were responsible for the original study design, conduct and analysis. TB was responsible for the design of the hospital based reactivation care program to be evaluated. JvW was responsible for the design of the qualitative part of the study (e.g. audit, fidelity study and other qualitative measures). All authors contributed to writing and critically reviewing the manuscript and approved its final version.

## Pre-publication history

The pre-publication history for this paper can be accessed here:

http://www.biomedcentral.com/1471-2318/11/36/prepub
